# Construction of immunogenic cell death-related molecular subtypes and prognostic signature in colorectal cancer

**DOI:** 10.1515/med-2023-0836

**Published:** 2023-11-09

**Authors:** Chun Yu, Weixuan Yang, Li Tian, Yue Qin, Yaoyao Gong, Wenfang Cheng

**Affiliations:** Department of Gastroenterology, Jiangsu Province Hospital and Nanjing Medical University First Affiliated Hospital, Nanjing 210029, China; Department of Gastroenterology, The Fifth People’s Hospital of Huai’an, Huai’an 223300, China; Department of Gastroenterology, Zigong Fourth People’s Hospital, Zigong 643000, China

**Keywords:** colorectal cancer, immunogenic cell death, molecule subtypes, prognostic model, immunotherapy

## Abstract

Immunotherapy is a promising treatment for advanced colorectal cancers (CRCs). However, immunotherapy resistance remains a common problem. Immunogenic cell death (ICD), a form of regulated cell death, induces adaptive immunity, thereby enhancing anti-tumor immunity. Research increasingly suggests that inducing ICD is a promising avenue for cancer immunotherapy and identifying ICD-related biomarkers for CRCs would create a new direction for targeted therapies. Thus, this study used bioinformatics to address these questions and create a prognostic signature, aiming to improve individualized CRC treatment. We identified two ICD -related molecular subtypes of CRCs. The high subtype showed pronounced immune cell infiltration, high immune activity, and high expression of human leukocyte antigen and immune checkpoints genes. Subsequently, we constructed and validated a prognostic signature comprising six genes (*CD1A*, *TSLP*, *CD36*, *TIMP1*, *MC1R*, and *NRG1*) using random survival forest analyses. Further analysis using this prediction model indicated that patients with CRCs in the low-risk group exhibited favorable clinical outcomes and better immunotherapy responses than those in the high-risk group. Our findings provide novel insights into determining the prognosis and design of personalized immunotherapeutic strategies for patients with CRCs.

## Graphical abstract

**Figure:**
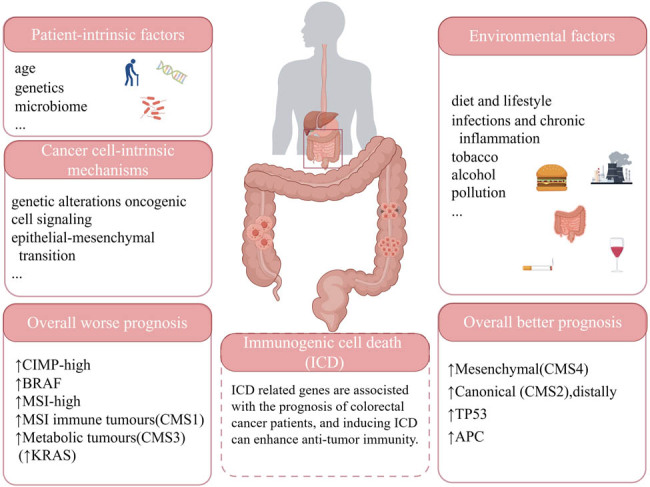

## 1 Introduction

Colorectal cancer (CRC) is currently the third most commonly diagnosed and the second most deadly cancer worldwide [1]. According to the Global Cancer Observatory, approximately 1.93 million (10%) new cases and 0.94 million (9.4%) cancer deaths due to CRC were recorded in 2020 [1]. The most common pathologic staging system for CRC is the 8th edition of the American Joint Committee Cancer Tumor Node Metastasis system, which ranges from stage I to IV, with stage 0 being the earliest stage of CRC [2]. Surgery and postoperative chemotherapy/radiotherapy have improved the prognosis of patients with early-stage CRC; however, treating advanced CRC remains a challenge [3,4]. The mechanisms underlying the development and progression of CRC are complex and have not been fully elucidated. Known factors include patient-intrinsic factors (e.g., age, genetics, and the microbiome), environmental factors (e.g., diet and lifestyle, infections and chronic inflammation, tobacco, alcohol, and pollution), and cancer cell-intrinsic mechanisms (e.g., genetic alterations, oncogenic cell signaling, and the epithelial–mesenchymal transition) [1,5]. Immunotherapy is a valuable treatment option for those with late-stage CRC. For example, immune checkpoint inhibitors received regulatory approval in 2017 for treating mismatch-repair-deficient (dMMR) or microsatellite instability-high metastatic CRC [6,7,8]. However, immunotherapy resistance remains common. Therefore, identifying biomarkers indicative of the immunotherapy response and prognosis of patients with CRC is vital.

According to the 2018 Nomenclature Committee on Cell Death classifications, immunogenic cell death (ICD) is a particular form of regulated cell death that can trigger an adaptive immune response [9,10]. Cells undergoing ICD are characterized by the release of damage-associated molecular patterns, including heat shock proteins 70 and 90, calreticulin, high-mobility group protein B1, and ATP [11,12]. Mounting studies have shown that treatment-driven ICD enhances the treatment effects of conventional radiotherapy and chemotherapy by inducing effective immune responses [13,14]. Moreover, studies have found that ICD combined with immune checkpoint inhibitors improves the anti-tumor immune effect [15,16]. Since ICD is inducible with cytotoxic drugs, it offers a potential approach for cancer immunotherapy [17].

In recent years, many preclinical studies have investigated the molecular mechanisms of ICD, but few studies have evaluated the possibility of ICD in a clinical context. Therefore, this study recognized ICD-associated subtypes of CRC and differences between subtypes in terms of prognosis, immune landscape, and somatic mutation. And then established a prognostic signature based on ICD-related gene expression. Overall, we aimed to improve immunotherapy response predictions for patients with CRC and provide new strategies for individualized therapy selection.

## 2 Materials and methods

### 2.1 Data source

The Cancer Genome Atlas (TCGA)-colon adenocarcinoma (COAD) and TCGA-rectal adenocarcinoma (READ) RNA sequencing data and matching clinical information were downloaded from the Genomic Data Commons (GDC) data portal (https://portal.gdc.cancer.gov/) to create the training set. Principal component analysis of the rectal and colon samples revealed no significant differences, and merging the samples did not require adjustments [18]. Thus, the COAD and READ samples were combined into a TCGA-CRC cohort. The GSE17538 dataset was downloaded from the Gene Expression Omnibus (GEO) database as a validation set [19]. Additionally, we obtained the list of ICD-related genes from the GeneCards database (http://www.genecards.org/).

### 2.2 Consensus clustering

We performed a cluster analysis to identify ICD-related molecule subtypes using the “ConsensusClusterPlus” package in R software (R Core Team, Vienna, Austria), as previously reported [20]. Subsequently, to ensure the stability of the results, we examined an optimal cluster size between *k* = 2–9 and repeated the analysis 1,000 times. The cluster map was created using the “pheatmap” tool in R.

### 2.3 Differentially expressed (DE) gene and functional enrichment analyses

DE genes were identified utilizing the “Limma” package in R, and the false discovery rate (FDR) was used to correct for false-positive results. The filter criteria were FDR < 0.05 and |logFoldchange (FC)| > 1. The DE genes were then subjected to Gene Ontology (GO) enrichment and Kyoto Encyclopedia of Genes and Genomes (KEGG) pathway analyses using the R package “clusterProfiler” [21]. GO terms and KEGG pathways were considered significantly enriched when the corrected *p*-value (i.e., the *q*-value) was <0.05.

### 2.4 Gene set enrichment analysis (GSEA)

We conducted a GSEA to assess functional variations between ICD-high and ICD -low clusters; c2kegg and c5go were used as the reference gene sets. The screening conditions were *p*-values of <0.05, FDR *q*-values of  <0.1, and |normalized enrichment scores (NESs)| of >1.5.

### 2.5 Somatic mutation analysis

We downloaded the COAD and READ somatic mutation data from the GDC data portal. The “Maftools” package in R was used to create waterfall plots and visualize the mutated genes, as previously reported [22].

### 2.6 Immune microenvironment differences between ICD high and low cohorts

The estimate score, immune score, stromal score, and tumor purity were calculated for each CRC sample using the ESTIMATE algorithm through the “estimate” package in R [18]. We then utilized the CIBERSORT algorithm to evaluate the relative proportions of 22 kinds of infiltrating immune cells in each sample, as previously reported [23]. Subsequently, we compared the expression levels of the immune checkpoint and human leukocyte antigen (HLA) genes between the two clusters.

### 2.7 Establishing and validating an ICD-related prognostic risk signature

Univariate Cox regression analysis and random survival forest-variable hunting algorithm (a powerful ensemble algorithm based on machine learning) were used for gene selection and model construction. First, a univariate Cox analysis was used to identify prognosis-related ICD genes. Then, using the R package “randomForestSRC,” we set the parameter ntree at 1,000 to predict the significant ICD genes from preliminary screened candidates. Then, based on the multivariate Cox analysis, Kaplan–Meier (KM) tests were performed for several gene combination signatures, and *p*-values calculated by KM analyses were sorted for comparisons and determining the optimal combination. Receiver operating characteristic (ROC) curves were used to assess the prognostic accuracy of the risk signature. The independent GSE17538 data set was used to verify the model’s stability. Finally, after identifying independent prognostic factors of CRC, we constructed a nomogram by using the “rms” package in R and evaluated its performance.

### 2.8 Immunotherapy response predictions

The tumor immune dysfunction and exclusion (TIDE) algorithm is a computing architecture that integrates data on two tumor immune escape mechanisms; we used this algorithm to predict the response to immune checkpoint therapy [24]. The TIDE database (http://tide.dfci.harvard.edu/) was used to calculate the TIDE score for each TCGA-CRC sample; then, we compared the relevance between the risk score and immunotherapy response.

### 2.9 Statistical analysis

R v4.1.2 (https://cran.r-project.org/) or GraphPad Prism software was used to analyze data. Continuous variables were reported as means and standard deviations and compared using a Student’s *t*-tests with *p*-values. Data were visualized using R v4.1.2, GraphPad Prism, SangerBox (http://vip.sangerbox.com), Figdraw (https://www.figdraw.com), and Hiplot (https://hiplot.com.cn) software.

## 3 Results

### 3.1 Differential ICD-associated genes and biological functions

**Figure 1:**
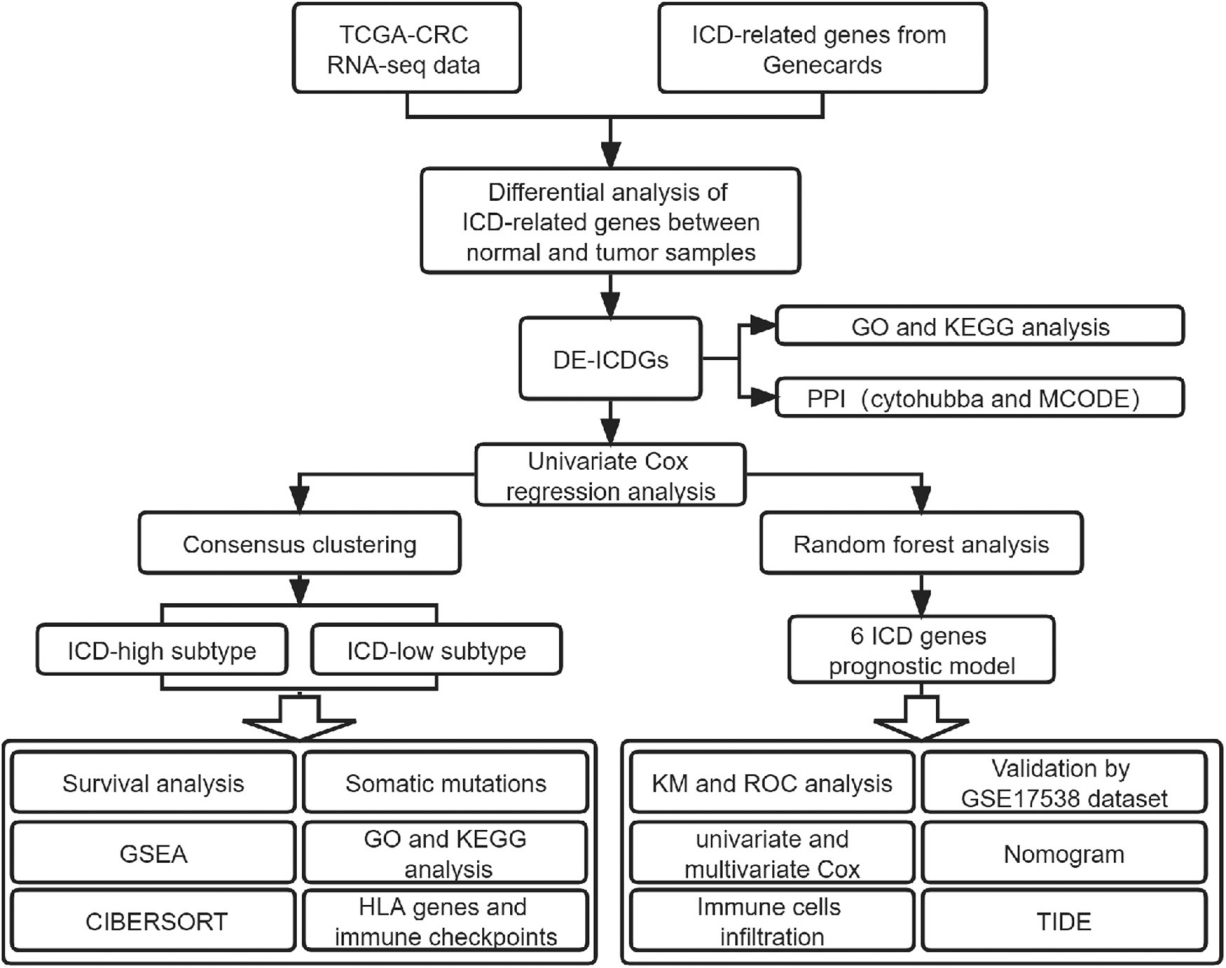
Study workflow diagram. CRC: colorectal cancer; DE: differentially expressed; GO: Gene Ontology; GSEA: gene set enrichment analysis; HLA: human leukocyte antigen; ICD: immunogenic cell death; KEGG: Kyoto Encyclopedia of Genes and Genomes; KM: Kaplan–Meier; PPI: protein–protein interaction; ROC: receiver operating characteristic; TCGA: The Cancer Genome Atlas; TIDE: tumor immune dysfunction and exclusion.

**Figure 2:**
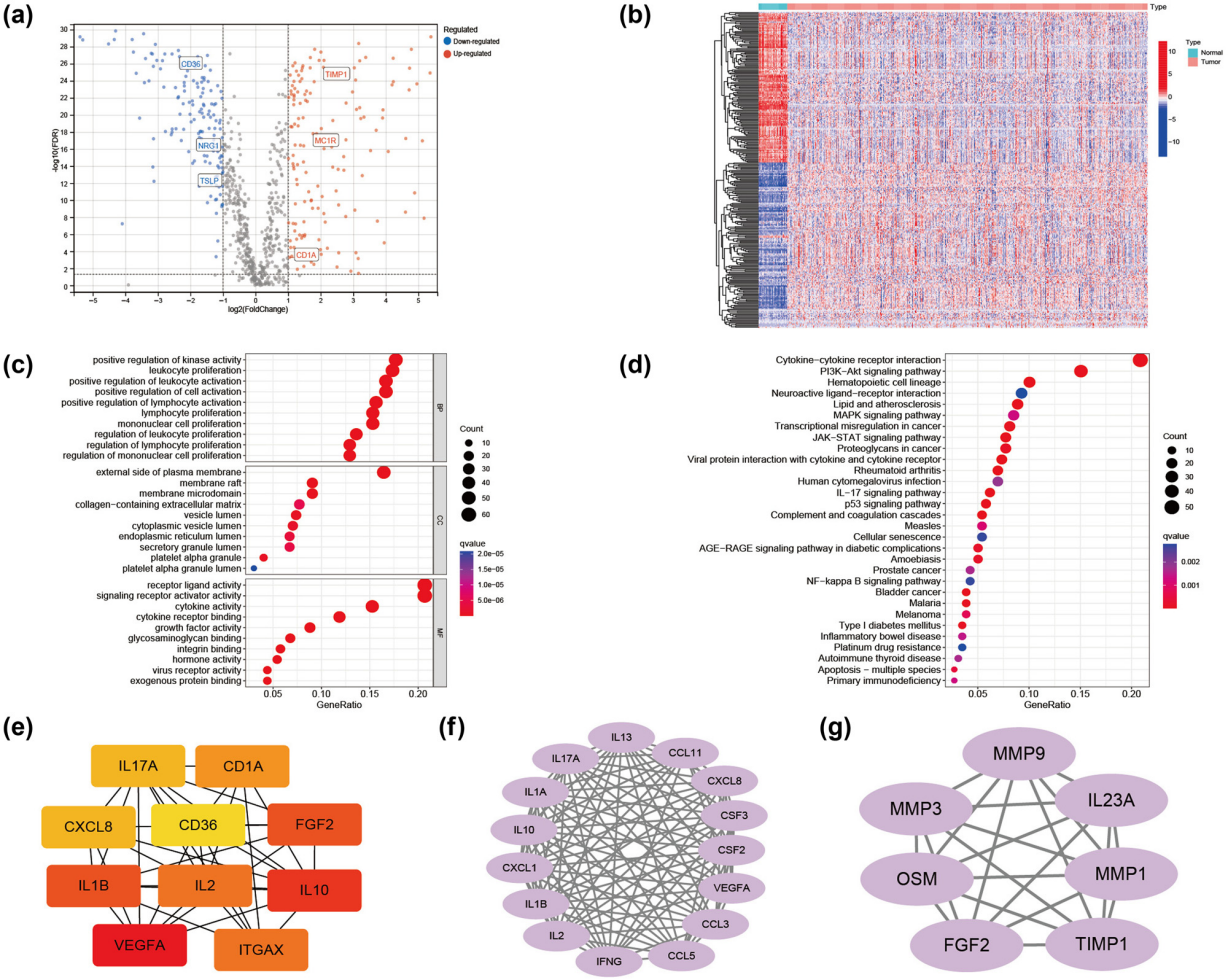
DE-ICD genes and a functional enrichment analysis of the DE-ICD genes. (a) Volcano plot of 154 up-regulated and 144 down-regulated ICD genes in the TCGA-CRC dataset (FDR < 0.05 and |logFoldchange| > 1). (b) Heatmap illustrating the DE-ICD genes in normal and CRC samples. (c) The top 30 enriched terms in GO enrichment analysis of the DE-ICD genes. (d) The top 30 enriched terms in the KEGG analysis. (e) The top 10 hub genes. (f and g) Modules obtained from the “MCODE” plugin. CRC: colorectal cancer; DE: differentially expressed; FDR: false discovery rate; ICD: immunogenic cell death; TCGA: The Cancer Genome Atlas.

**Figure A1:**
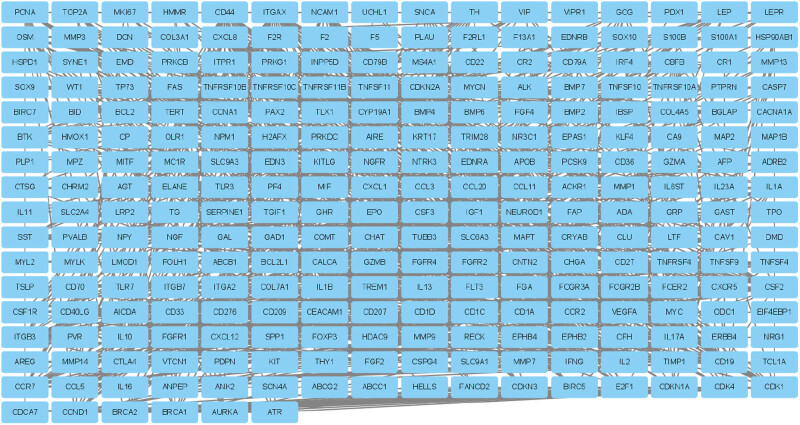
Protein-protein interactions network of the differentially expressed (DE) - immunogenic cell death (ICD) genes.

Figure 1 presents a workflow overview. First, 859 ICD-related genes were obtained from the GeneCards database; they were screened based on protein-coding genes and relevance scores larger than the median. Then, we identified 298 DE-ICD genes; 154 were up-regulated, and 144 were down-regulated based on a differential analysis between tumor and normal samples in TCGA-CRC (Figure 2a and b). GO and KEGG analyses indicated that the DE-ICD genes were primarily enriched in the activation and regulation of inflammatory cells, activities associated with immunity, and cancer-related biological processes and signaling pathways (Figure 2c and d). To further understand the connections among these DE-ICD genes, we constructed a protein-protein interaction network using the STRING database (https://string-db.org), which was visualized usingCytoscape (Figure A1). Figure 2e–g presents the top ten hub genes and the two most significant modules identified using the CytoHubba and MCODE Cytoscape plugins.

### 3.2 ICD-related subtypes

**Figure 3:**
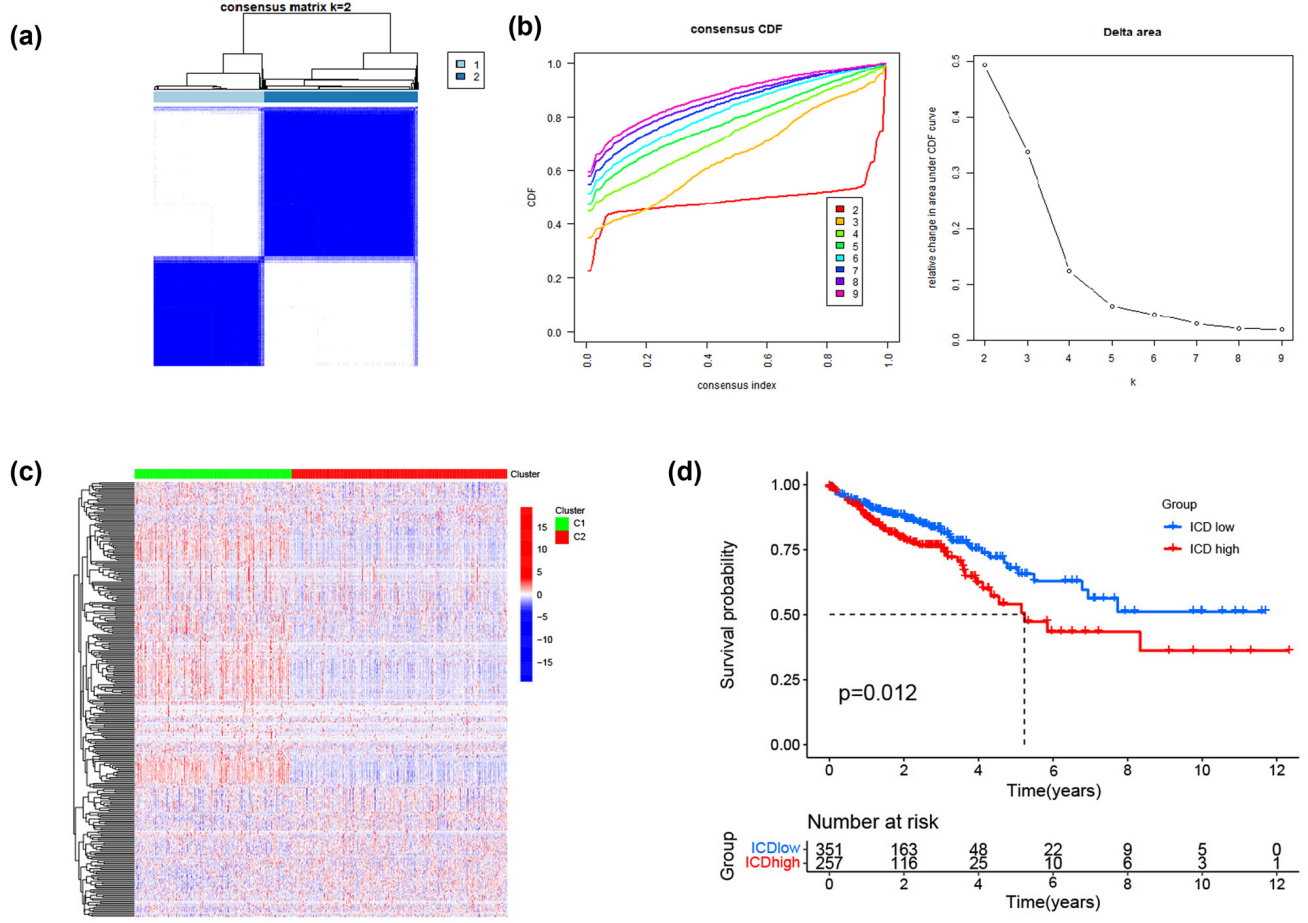
Identification of ICD-associated subtypes by consistent clustering. (a) Consensus clustering matrix when *k* = 2. (b) Relative change in the area under the cumulative distribution function curve for *k* = 2–9. (c) Heatmap of ICD-related gene expressions between the ICD-high and ICD-low subtypes. (d) KM curves of OS in ICD-high and ICD-low subtypes. CDF: cumulative distribution function; ICD: immunogenic cell death.

We determined the ICD-related clusters of TCGA-CRC through consensus clustering. Two subgroups were identified from the univariate analysis based on the prognosis-related genes (Figure 3a and b). DE-ICD gene expression levels were compared between the two clusters; cluster C1 was defined as the ICD-high group, and cluster C2 as the ICD-low group (Figure 3c). Then, a survival analysis between the two groups was performed, resulting in different clinical outcomes. The ICD-low and ICD-high subtypes were linked to favorable and unfavorable prognoses, respectively (Figure 3d).

### 3.3 Signal pathways, somatic mutations, and tumor microenvironment differences between the ICD subtypes

**Figure 4:**
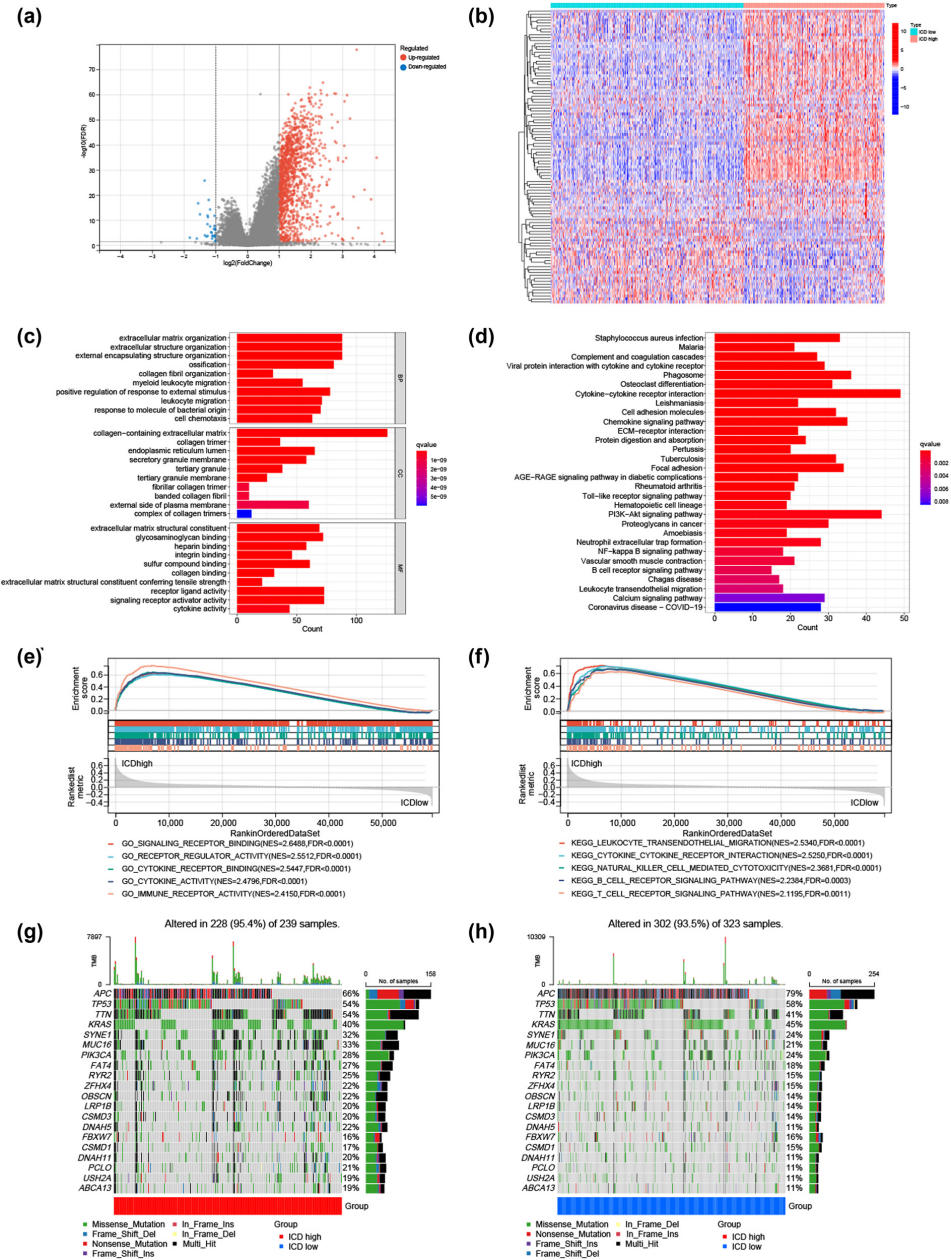
Analyses of DE genes, signaling pathways, and somatic mutations between the high and low ICD subtypes. (a) Volcano plot of 1,302 dysregulated genes and (b) a heatmap presenting the top 50 genes ordered based on the log foldchange values between the two subtypes. (c) The top 10 enriched terms in GO enrichment analysis and (d) the top 30 enriched terms in KEGG analysis of the genes up-regulated in the ICD-high subtype. GSEA between the ICD- (e) high and (f) low subtypes. The most frequently mutated genes in the ICD- (g) high and (h) low subtypes. DE: differentially expressed; ICD: immunogenic cell death.

Further comparison of the low and high ICD subtypes identified 1,302 dysregulated genes (Figure 4a); Figure 4b presents the top 50 genes ordered by the logFC values. GO enrichment and KEGG pathway analyses were performed using the genes up-regulated in the ICD-high subtype. These genes were enriched in biological functions and signaling pathways, including leukocyte migration, signaling receptor activator activity, cytokine activity, cytokine–cytokine receptor interaction, B-cell receptor, and the PI3K–Akt signaling pathway (Figure 4c and d). Several immune-related pathways were also associated with the ICD-high subtype, including natural killer cell-mediated cytotoxicity (NES = 2.3681, FDR < 0.0001), immune receptor activity (NES = 2.4150, FDR < 0.0001), T-cell receptor signaling pathway (NES = 2.1195, FDR = 0.0011), and B-cell receptor signaling pathway (NES = 2.2384, FDR = 0.0003), identified by GSEA (Figure 4e and f).

Furthermore, we explored somatic mutations between the two subtypes. *APC*, *TP53*, *TTN*, *KRAS*, and *SYNE1* were the top five genes with the highest mutation frequencies in both the high and low ICD subtypes, but their relative frequencies differed in each group (Figure 4g and h).

**Figure 5:**
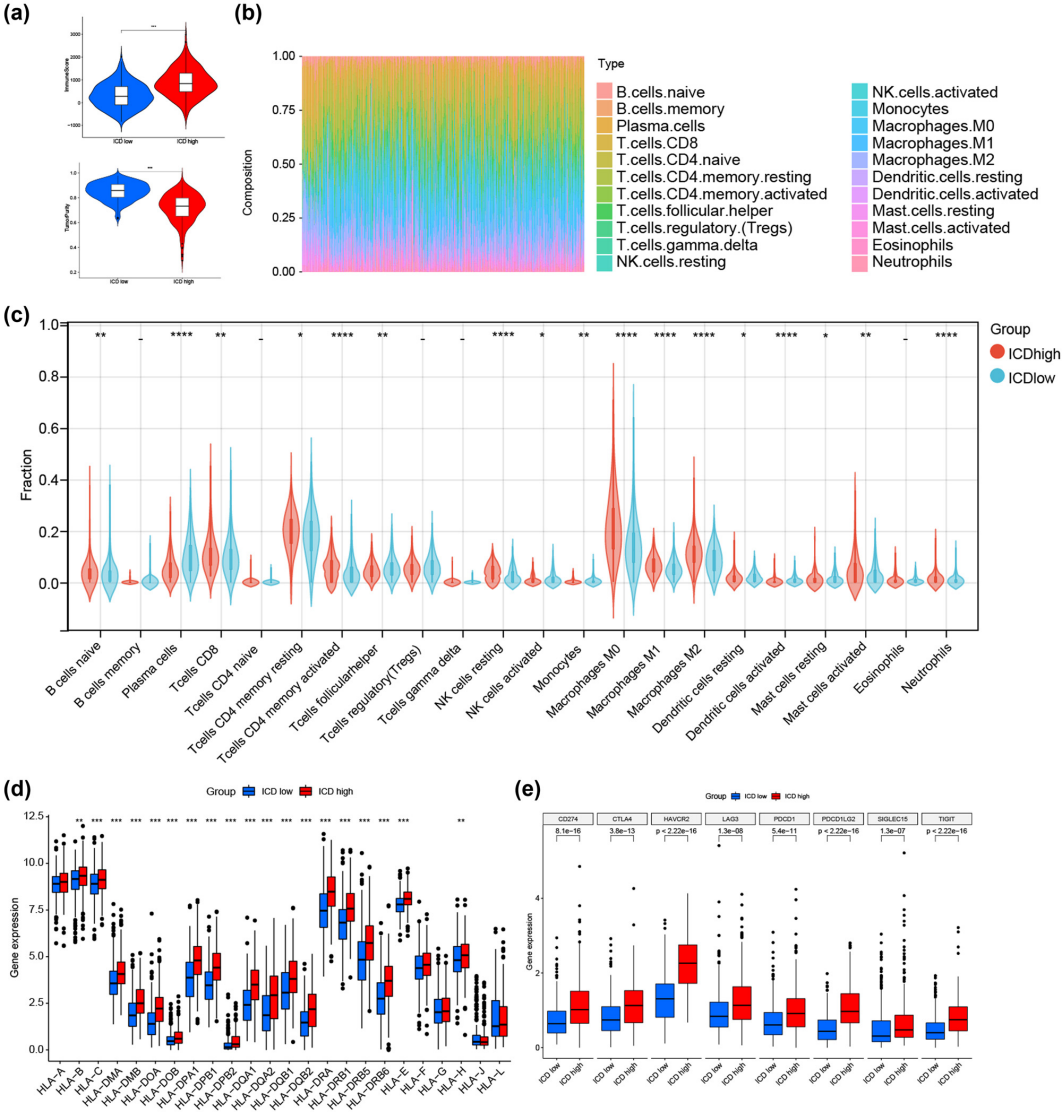
Tumor microenvironment analyses between the high and low ICD subtypes. (a) Violin plots of immune and tumor purity scores. (b) Relative proportion of immune infiltration in each CRC sample obtained from TCGA. (c) Immune cell infiltration differences between the high and low ICD subtypes. DE of (d) multiple HLA genes and (e) immune checkpoint genes between the high and low ICD subtypes. *p < 0.05, **p < 0.01, ***p < 0.001, and ****p < 0.0001. CD: cluster of differentiation; DE: differential expression; ICD: immunogenic cell death; NK: natural killer.

Increasing evidence has demonstrated that ICD is important in activating anti-tumor immune responses. Thus, we dissected the differences between the tumor microenvironment in the high and low ICD subtypes. The ESTIMATE algorithm showed that the ICD-high subtype had a higher immune score and lower tumor purity than the ICD-low subtype (Figure 5a). Subsequently, the CIBERSORT algorithm was used to analyze the differentiation in infiltration of immune cells between the two subtypes; Figure 5b presents the results of each TCGA-CRC sample. Patients in the ICD-high subtype had higher percentages of CD8 T cells, naive B cells, activated memory CD4 T cells, macrophages, and neutrophils than those in the ICD-low subtype (Figure 5c). In addition, most HLA and immune checkpoint genes were up-regulated in the ICD-high subtype (Figure 5d and e). Therefore, the ICD-high subtype may be related to the immune-hot phenotype.

### 3.4 Establishing and validating an ICD-related prognostic risk model

**Figure 6:**
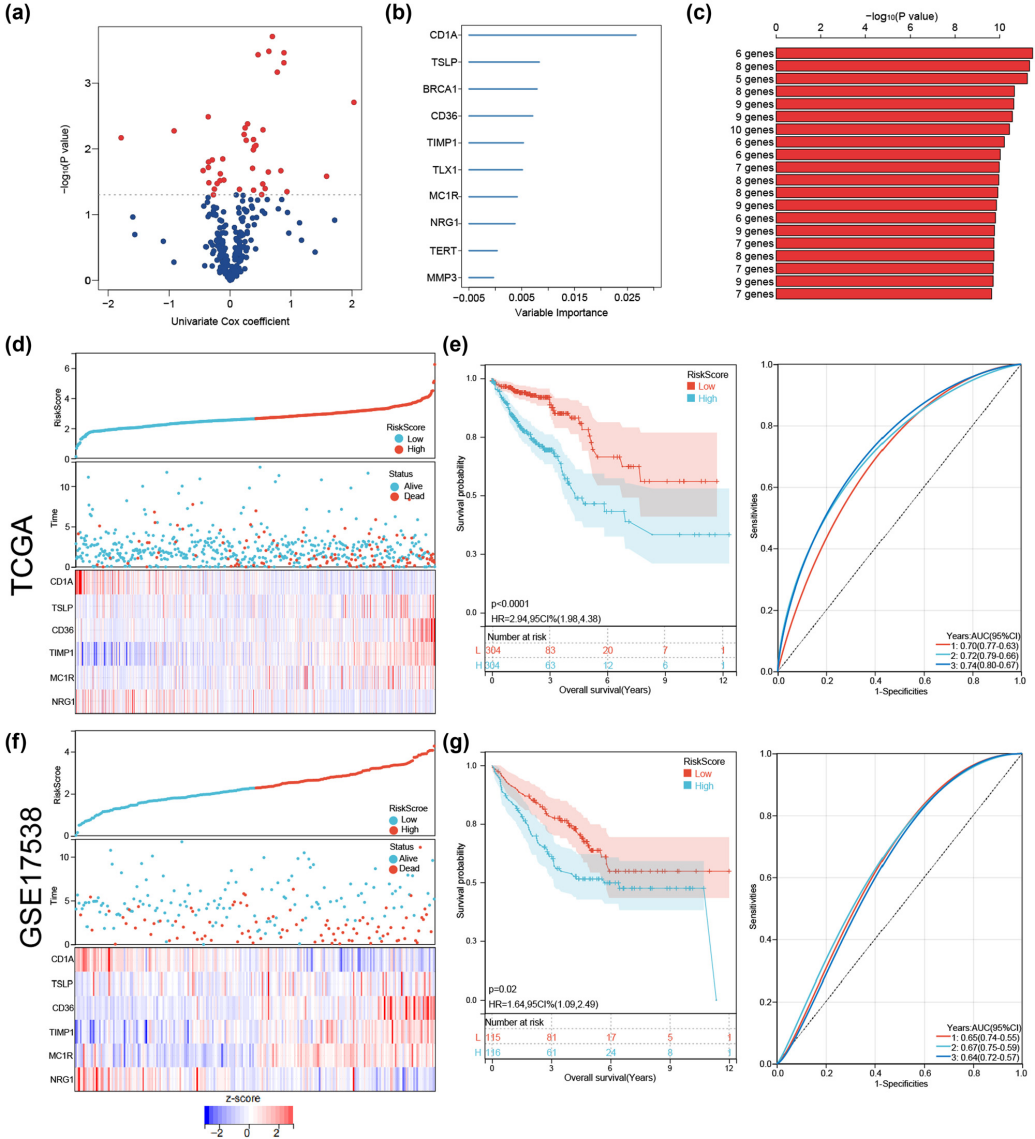
Construction and validation of the ICD risk signature. (a) Volcano plot of 41 genes associated with OS identified by univariate Cox analysis. (b) Top 10 genes ranked by importance in the random forest analysis. (c) Combinations of genes as a prognostic model were ordered based on the −log10 (*p*-value). Distribution of individual risk scores and survival statuses and heatmaps of the prognostic six-gene signature demonstrate significant differences between the high-risk and low-risk groups in (d) TCGA-CRC and (f) GSE17538 cohorts. KM and ROC analysis in (e) TCGA-CRC and (g) GSE17538 cohorts. AUC: area under the curve; CI: confidence interval; CRC: colorectal cancer; HR: hazard ratio; ICD: immunogenic cell death; ROC: receiver operating characteristic; TCGA: The Cancer Genome Atlas.

To better predict the prognosis of patients with CRC, we built a prognostic model comprising six ICD genes. In the univariate Cox analysis of 298 DE-ICD genes, 41 were associated with overall survival (OS) in the TCGA-CRC cohort (Figure 6a). These genes were reserved for further analysis, and a six-gene prognostic risk model was established in the random survival forest analysis (Figure 6b and c). The risk score formula was: risk score = (−1.2621 × *CD1A*) + (0.7546 × *TSLP*) + (0.4412 × *CD36*) + (0.4615 × *TIMP1*) + (0.3879 × *MC1R*) + (−0.9271 × *NRG1*).

Patients in the TCGA-CRC cohort were stratified into high- and low-risk groups based on the median risk score value, and the association between survival status and risk score was visualized. The number of deceased patients in the high-risk group was considerably higher than that in the low-risk group. Figure 6d illustrates the expression level differences of the six genes between the two groups.

KM analysis indicated that patients with CRC and low-risk scores in the TCGA cohort had better OS times than those with high-risk scores. The predictability and sensitivity of the risk score model were validated by ROC analysis (Figure 6e). The areas under the curve of the prognostic model at 1, 2, and 3 years in the TCGA dataset were 0.70, 0.72, and 0.74, respectively, demonstrating its excellent predictive model performance. As an independent external validation set, GSE17538 was used to further evaluate the efficiency of the constructed prognostic model, which also confirmed the model’s good performance (Figure 6f and g).

**Figure 7:**
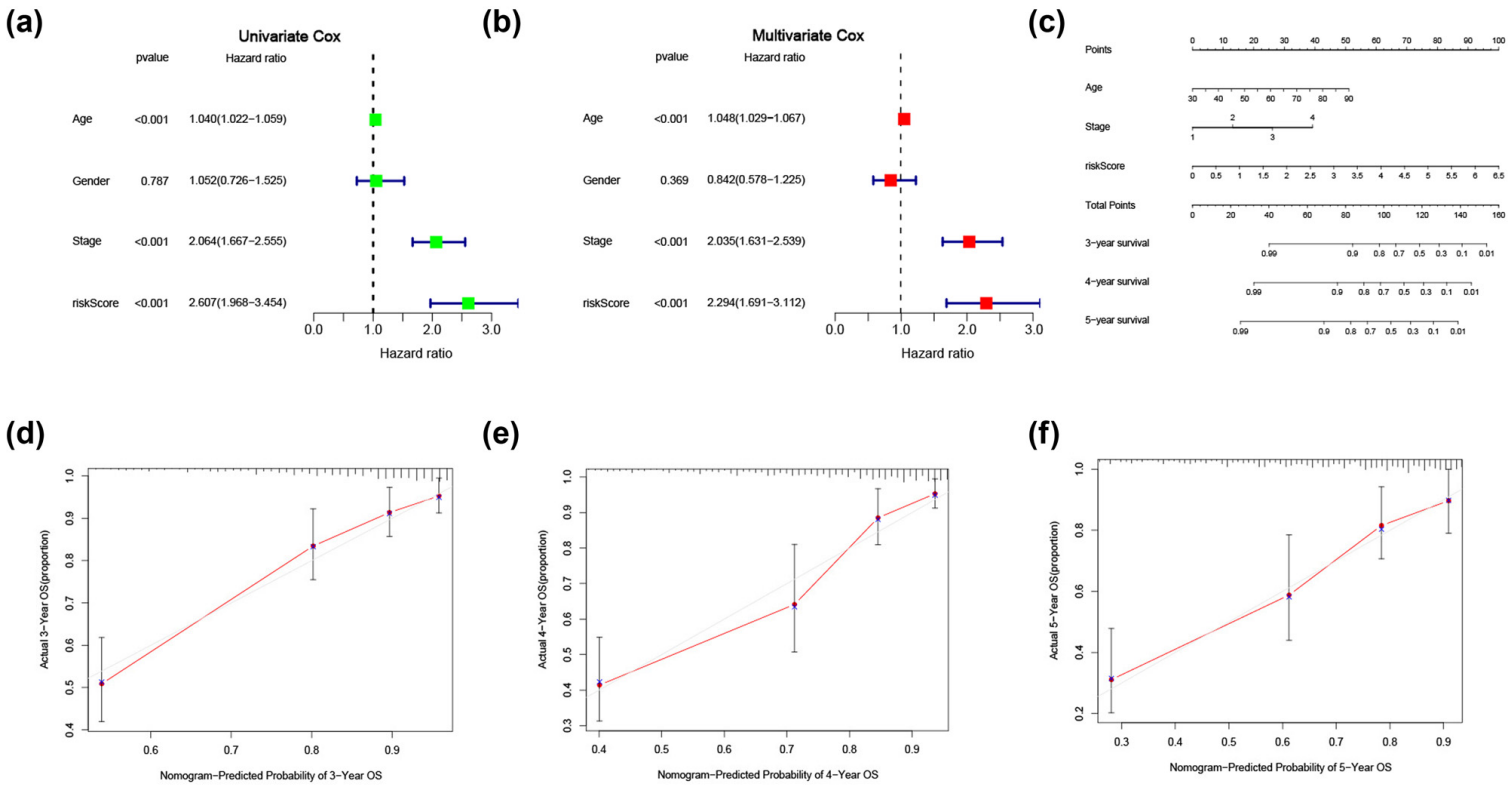
Construction and internal validation of a predictive nomogram of OS for patients with CRCs. (a) Univariate and (b) multivariate Cox analyses evaluating the independent prognostic value of the risk signature in patients with CRC. (c) Nomogram based on age, stage, and signature risk score. Calibration plots of the nomogram for predicting the probability of (d) 3-, (e) 4-, and (f) 5-year survival. CRC: colorectal cancer; OS: overall survival.

In addition, we performed univariate and multivariate Cox regression analyses, finding that age, tumor stage, and risk score were independent prognostic factors in patients with CRC (Figure 7a and b). Subsequently, a nomogram including the risk score, stage, and age was created (Figure 7c). Calibration plots revealed that the predicted probability of OS probabilities at 3, 4, and 5 years were highly concordant with the actual OS (Figure 7d–f).

### 3.5 Correlations between tumor immune cell infiltration and immunotherapy response and the ICD risk signature

**Figure 8:**
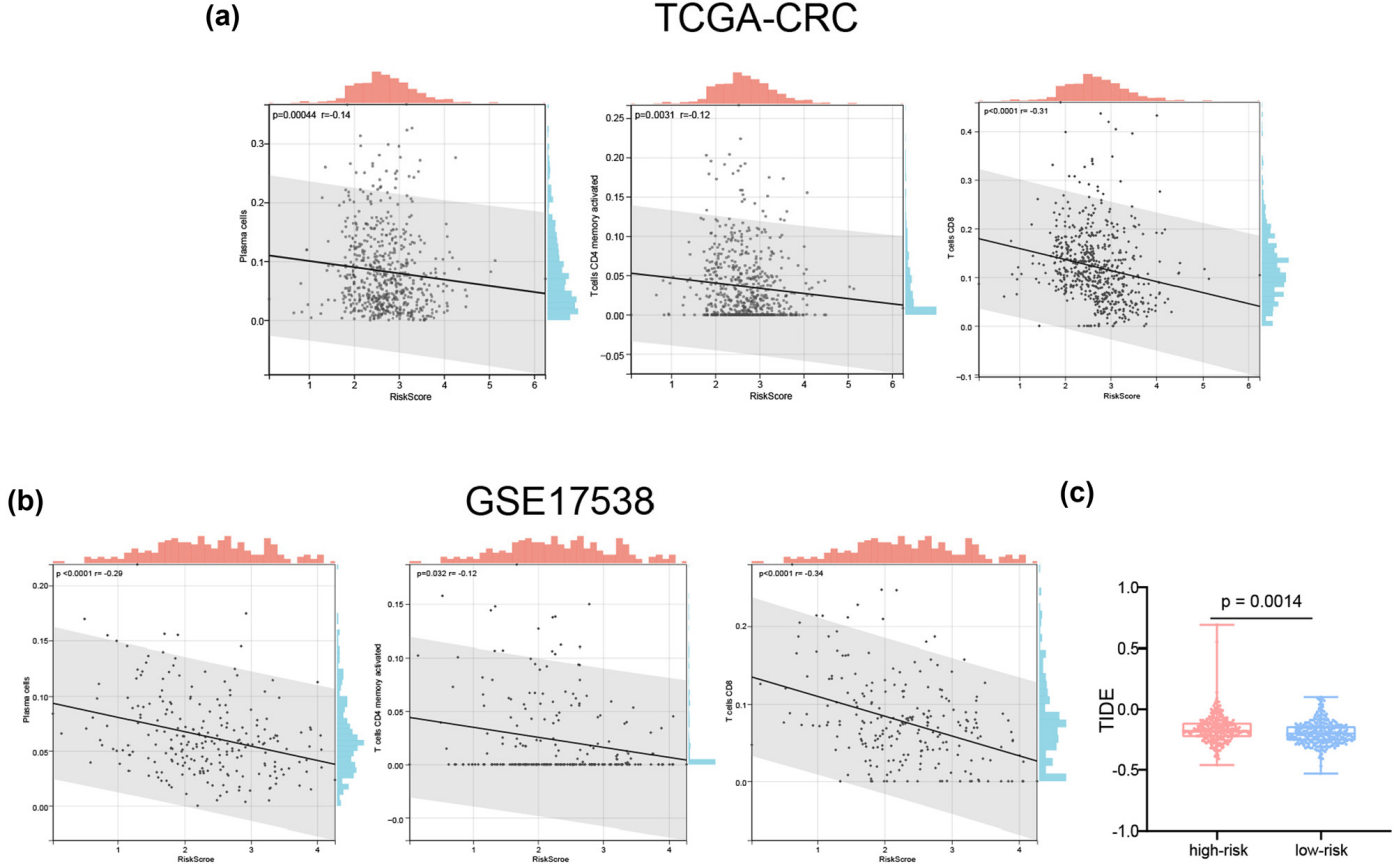
Correlations between the risk signature and tumor microenvironment. Scatter plots present the relationships between the risk score and the infiltration of plasma cells, activated memory CD4 T cells, and CD8 T cells in the (a) TCGA-CRC and (b) GSE17538 datasets. (c) The TIDE scores in the high- and low-risk groups. CRC: colorectal cancer; TCGA: The Cancer Genome Atlas; TIDE: tumor immune dysfunction and exclusion.

ICD plays a considerable role in anti-tumor immune response. Thus, we assessed the connections between the risk scores and infiltrating immune cells. The risk scores of samples in the TCGA-CRC cohort samples negatively correlated with activated memory CD4 T cells, CD8 T cells, and plasma cells (Figure 8a). Similar results were obtained using the GSE17538 dataset (Figure 8b).

We then used the TIDE algorithm to evaluate the predictive power of the ICD-related risk score in response to immunotherapy. Compared with those in the high-risk group, patients in the low-risk group were associated with low TIDE scores in the TCGA cohort (Figure 8c), demonstrating that patients with CRC and low-risk scores should benefit from immunotherapy more than those with high-risk scores.

## 4 Discussion

In recent years, immunotherapy has become an important therapeutic method for patients with metastatic CRC. However, owing to the low response rate and side effects, cancer immunotherapy is effective in only a small subset of patients; however, inducing ICDs may mitigate these challenges [25]. As a new form of regulatory cell death, ICD has been found to induce adaptive immunity, thereby enhancing anti-tumor immunity. Thus, identifying ICD-related biomarkers may help distinguish CRC patients who could benefit from immunotherapy, similar to ICD markers in melanoma [26], head and neck squamous cell carcinoma [27], and neuroblastoma [28]. Furthermore, a recent study reported a breast cancer prognostic risk model comprising seven ICD genes for estimating the prognosis of patients with breast cancer and their response to immunotherapy [29], similar to our research aim.

To our knowledge, this study is the first to describe the role of ICD genes in tumor microenvironment differentiation and CRC prognosis. We identified two distinct subtypes of patients with CRC based on the expression of ICD-related genes, which correlated with diverse infiltration levels of various immune cells and different survival prognoses. The ESTIMATE algorithm showed that patients with the ICD-high subtype had high immune scores, low tumor purity, and increased immune cell infiltration. Furthermore, the up-regulated genes in the ICD-high subtype were enriched in leukocyte migration, signaling receptor activator activity, cytokine activity, and cytokine–cytokine receptor interaction; immune-related signaling pathways were also significantly enriched. Most HLA genes and immune checkpoint genes were also up-regulated in the ICD-high group. Some have reported that tumors can escape immune surveillance by reducing the expression of major histocompatibility complex (MHC) molecules [30]. Immune checkpoint inhibitors have also become a promising treatment method in cancer immunotherapy [31]. Our results demonstrated that the ICD-high subgroup was related to the immune-hot phenotype.

Moreover, we constructed and validated a six-gene (*CD1A*, *TSLP*, *CD36*, *TIMP1*, *MC1R*, and *NRG1*) prognostic risk signature. The risk score was associated with the immune context of the tumor microenvironment, and it has the potential ability to predict an immunotherapy response. TSLP is a pro-Th2 cytokine primarily expressed by epithelium cells and is thought to be a key cytokine linking innate and adaptive immune systems [32,33]. Many reports have noted that TSLP-dependent inflammatory Th2-type response and pro-tumorigenic functions play roles in pancreatic, breast, and cervical cancers [34,35,36]. TSLP also affects CRC progression by regulating the function of tumor-specific regulatory T cells [37]. CD36, a transmembrane glycoprotein receptor, is expressed in tumor, stromal, and immune cells and plays a significant role in regulating cell adhesion, immune response, metastasis, and angiogenesis in tumors [38,39]. Decreased CD36 expression might contribute to tumor cell evasion from the immune system and targeting CD36 may be a potential therapeutic strategy in cancer immunotherapy [40,41]. TIMP1 suppresses immune responses indirectly by regulating matrix metalloproteinase expression [42,43]; it also has an essential role in the development of gastrointestinal malignancies [44,45]. In patients with CRC, up-regulated TIMP1 expression is related to distant metastasis, vascular invasion, and lymphatic metastasis [46]. MC1R was initially described in cells of melanocytic origin but was later found on fibroblasts and most immune cells, indicating that it can affect innate and adaptive immunities [47,48]. Reports suggest that high MC1R expression might be significantly related to microsatellite instability and poor prognosis in CRC [49]. CD1A is an MHC class I-like molecule expressed by immune cells, including dendritic and Langerhans cells [50]. CD1A-positive dendritic cell infiltration is associated with favorable prognosis in patients with CRC [51], esophageal carcinoma [52], and breast cancer [53].

Our study has some limitations. Above all, the prognostic risk model was only validated on the GEO database, and prospective studies with more extensive sample numbers are needed to demonstrate the clinical value of the model. Furthermore, *in vivo* and *in vitro* biological experiments of the six genes in CRC are needed. Finally, this study only suggested a possible relationship between immune status and ICD subtypes or the risk model. Thus, we will collect sufficient samples in future studies to evaluate the value of this typing method and risk model in immunotherapy.

## 5 Conclusion

In this study, bioinformatics analyses of abnormally expressed ICD genes identified two distinct ICD subtypes in CRC: low and high. The ICD-low and ICD-high subtypes were associated with favorable and unfavorable prognoses, respectively. Moreover, we used ICD-related genes (*CD1A*, *TSLP*, *CD36*, *TIMP1*, *MC1R*, and *NRG1*) to construct and validate a prognostic risk signature of CRC; patients with low-risk scores had longer OS and might benefit from immunotherapy more than those with high-risk scores. This study highlights the predictive value of the ICD-related genes in CRC and immunotherapy response prognoses.
